# Pyroptosis in sepsis: Comprehensive analysis of research hotspots and core genes in 2022

**DOI:** 10.3389/fmolb.2022.955991

**Published:** 2022-08-11

**Authors:** Demeng Xia, Sheng Wang, Renqi Yao, Yuexue Han, Liyu Zheng, Pengyi He, Ying Liu, Lu Yang

**Affiliations:** ^1^ Luodian Clinical Drug Research Center, Shanghai Baoshan Luodian Hospital, Shanghai University, Shanghai, China; ^2^ Department of Emergency, Changhai Hospital, Naval Medical University, Shanghai, China; ^3^ Translational Medicine Research Center, Fourth Medical Center and Medical Innovation Research Division of the Chinese PLA General Hospital, Beijing, China; ^4^ Department of Clinical Medicine, Qing Dao University, Qing Dao, Shan Dong, China; ^5^ Institute of Translational Medicine, Shanghai University, Shanghai, China; ^6^ Clinical Research Center, Shanghai Baoshan Luodian Hospital, Shanghai University, Shanghai, China

**Keywords:** pyroptosis, sepsis, toll and nod signaling pathways, NLRP3 and immune organs, bibliometrics

## Abstract

Sepsis, a life-threatening disease caused by dysregulated host response to infection, is a major public health problem with a high mortality and morbidity rate. Pyroptosis is a new type of programmed cell death discovered in recent years, which has been proved to play an important role in sepsis. Nevertheless, there is no comprehensive report, which can help researchers get a quick overview and find research hotspots. Thus, we aimed to identify the study status and knowledge structures of pyroptosis in sepsis and summarize the key mechanism of pyroptosis in sepsis. The data were retrieved and downloaded from the WOS database. Software such as VOSviewer was used to analyze these publications. Key genes were picked out by using (https://www.genecards.org) and (http://www.bioinformatics.com). Then, Gene Ontology (GO) enrichment analysis and Kyoto Encyclopedia of Genes and Genomes (KEGG) pathway analysis were used to performed these key genes. From 2011 to 2021, a total of 299 papers met the search criteria, and the global interest in pyroptosis in sepsis measured by the value of (RRI) has started to increase since 2016. China ranked first in the number of publications, followed by the USA. The journal *Frontiers in Immunology* published the most relevant articles. Through keyword co-occurrence analysis, the high-frequency subject terms were divided into three clusters like “animal research”, “cell research,” and “molecular research” clusters. “mir,” “aki,” “monocyte,” and “neutrophil” were the newest keywords that may be the hotspot. In addition, a total of 15 genes were identified as hub genes. TNF, IL-1β, AKT1, CASP1, and STAT3 were highly expressed in lung tissues, thymus tissues, and lymphocytes. KEGG analysis indicated that pyroptosis may play a vital role in sepsis *via* the NOD, PI3K/AKT, and MAPK/JNK pathways. Through the quantitative analysis of the literature on pyroptosis in sepsis, we revealed the current status and hotspots of research in this field and provided some guidance for further studies.

## Introduction

Pyroptosis is a pro-inflammatory programmed cell death. In 2001, a review (Boise and Collins, 2001) summarized the discovery of a different pattern of cell death caused by caspase-1 during infection from traditional apoptosis and named this pattern “pyroptosis” (Cookson and Brennan, 2001). Pyroptosis occurs rapidly, which is characterized by membrane disruption and release of cytoplasmatic contents with a pronounced inflammatory response (Fink and Cookson, 2006). Martinon et al. (2002) reported the findings of inflammasome first in 2002, which elucidated the key question on the activation of the crucial constituents of the inflammasome. The inflammasome is a multi-protein complex that identifies infectious and non-infectious stimuli, inducing the activation of caspase-1 and caspase-4/5/11, resulting in the activation of gasdermin D, which will disrupt cell membrane integrity (Ding et al., 2016). At this time, a vast set of pro-inflammatory factors were released and initiated inflammatory responses, leading to a cytokine storm (Evavold et al., 2018).

Sepsis is defined as life-threatening organ dysfunction due to a dysregulated host response to infection (Vincent et al., 2014; Singer et al., 2016). Although there have been significant advances in the management of septic shock in recent decades, the morbidity and mortality rate of patients with sepsis remain high in the world, especially in developing countries (Vincent et al., 2006; Fleischmann et al., 2016; Liao et al., 2016; Rudd et al., 2020). Moreover, sepsis is also an important issue in public health with a high therapeutic cost and poor eventual prognosis (Iwashyna et al., 2010).

The inflammatory/immune response during sepsis can initially defend against invading pathogens, which minimizes tissue damage. However, uncontrolled inflammation leads to tissue damage and possibly organ failure (Nedeva et al., 2019). Over the last few decades, accumulating evidence demonstrates that pyroptosis mediated by inflammasome activation may be the key mechanism of sepsis (Esquerdo et al., 2017; Wang et al., 2018). Currently, the treatment strategies of inhibiting the caspase-1 signaling pathway and the caspase-11 pathway have been confirmed effectively in several sepsis models (Liu et al., 2017; Song et al., 2018). Thus, it is important to explore the pathophysiological mechanism of pyroptosis in sepsis.

By using literature databases and literature metrology characteristics as research objects, bibliometrics can analyze publications quantitatively and qualitatively (Share, 1976). Since bioinformatic analysis is widely used for interpreting highly dimensional biological data and sepsis is a complex disease involving multiple pathways, this method would be suitable to investigate the intricate mechanism of pyroptosis in sepsis. In this study, we summarized the publication trends of pyroptosis in sepsis. In addition, a systematic visual analysis was conducted using VOSviwer and Citespace software. Additionally, we investigated underlying mechanisms of pyroptosis in sepsis through Gene Ontology (GO) and Kyoto Encyclopedia of Genes and Genomes (KEGG) (Huang et al., 2007; Franceschini et al., 2013). Based on these results, we analyzed recent developments in pyroptosis in sepsis and trends since 2011, as well as identified key genes and mechanisms associated with pyroptosis, so as to provide new ideas or directions for future basic and clinical research.

## Materials and methods

The Web of Science is the largest and most comprehensive database of academic information available on the Internet. The search strategies were set as follows: (pyroptotic or pyroptosome or pyroptosis) AND (sepsis OR (septic shock) OR (endotoxemia) OR [SIRS OR (systemic inflammatory response syndrome)] OR (systematic inflammatory response syndrome) AND Language = English. The strategy of manual retrieval requires the overall evaluation of the research content of the article rather than just looking at the keywords. We make the initial judgment based on the abstract, and then make the second judgment based on the full text if there is any doubt. From the correlative publications, including titles, keywords, authors, publication dates, original countries and regions, institutions, references, H-index, and so on. Microsoft Excel 2016, VOSviewer version 1.6.12, and the Online Analysis Platform of Literature Metrology were used to analyze the country/region and institutional distribution, author contributions, core journals, keywords, and timeline viewer (Synnestvedt et al., 2005). The genes of pyroptosis and sepsis were comprehensively retrieved from GeneCards. The following keywords were used as search strategies: “pyroptosis” and “sepsis.” The results returned 235 and 2,775 entries, respectively. The two gene sets were intersected by using the online website (http://www.bioinformatics.com), resulting in a new intersection containing 139 key genes, which was used for subsequent analysis. The obtained gene sets were analyzed with Gene Ontology (GO) enrichment analysis and Kyoto Encyclopedia of Genes and Genomes (KEGG) pathway analysis. Following that, the network was displayed by Cytoscape software (Shannon et al., 2003). The genes were ranked according to their degree of connectivity with other genes, showing the map of the top 15 hub genes. For the five core genes in hub genes, we visualized and analyzed their expression levels in normal tissues.

## Results

**FIGURE 1:**
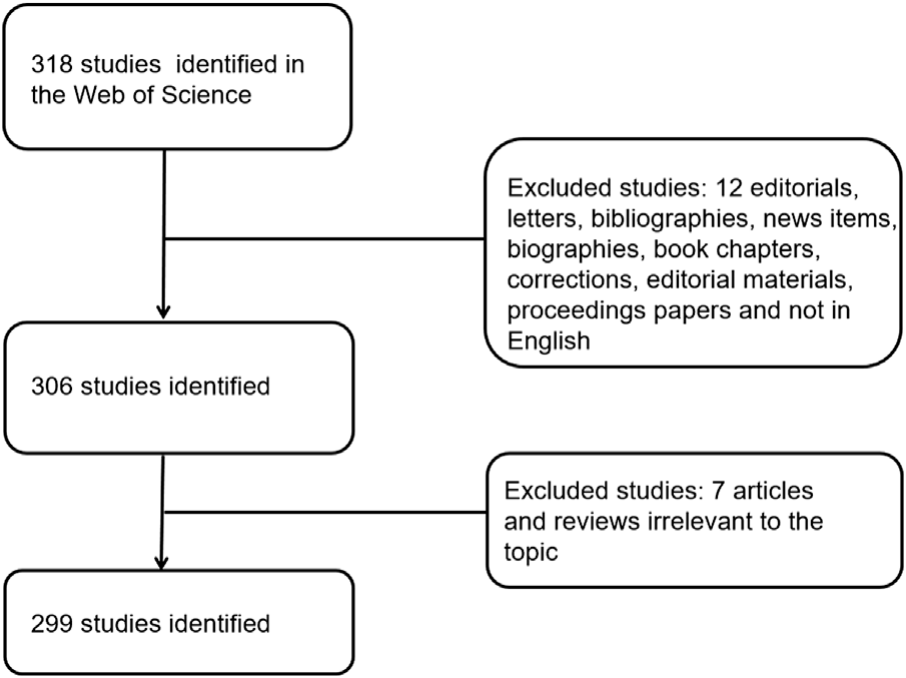
Flowchart of literature screening. The detailed process of screening and enrollment.

**FIGURE 2:**
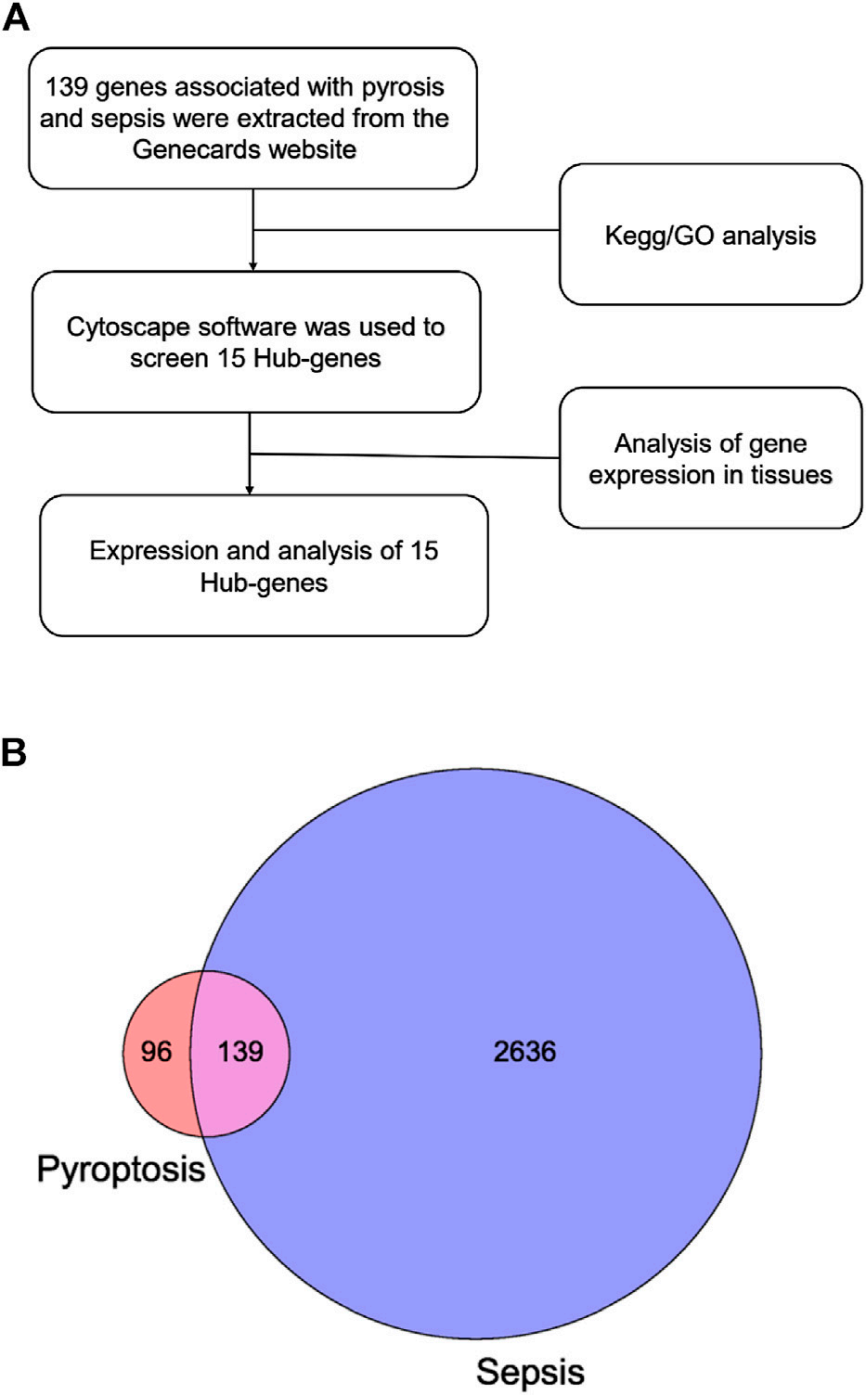
Strategy and results of bioinformatic analysis. **(A)** Flowchart of gene screening. **(B)** The Venn diagram shows the intersection between pyroptosis and sepsis genes.

**FIGURE 3:**
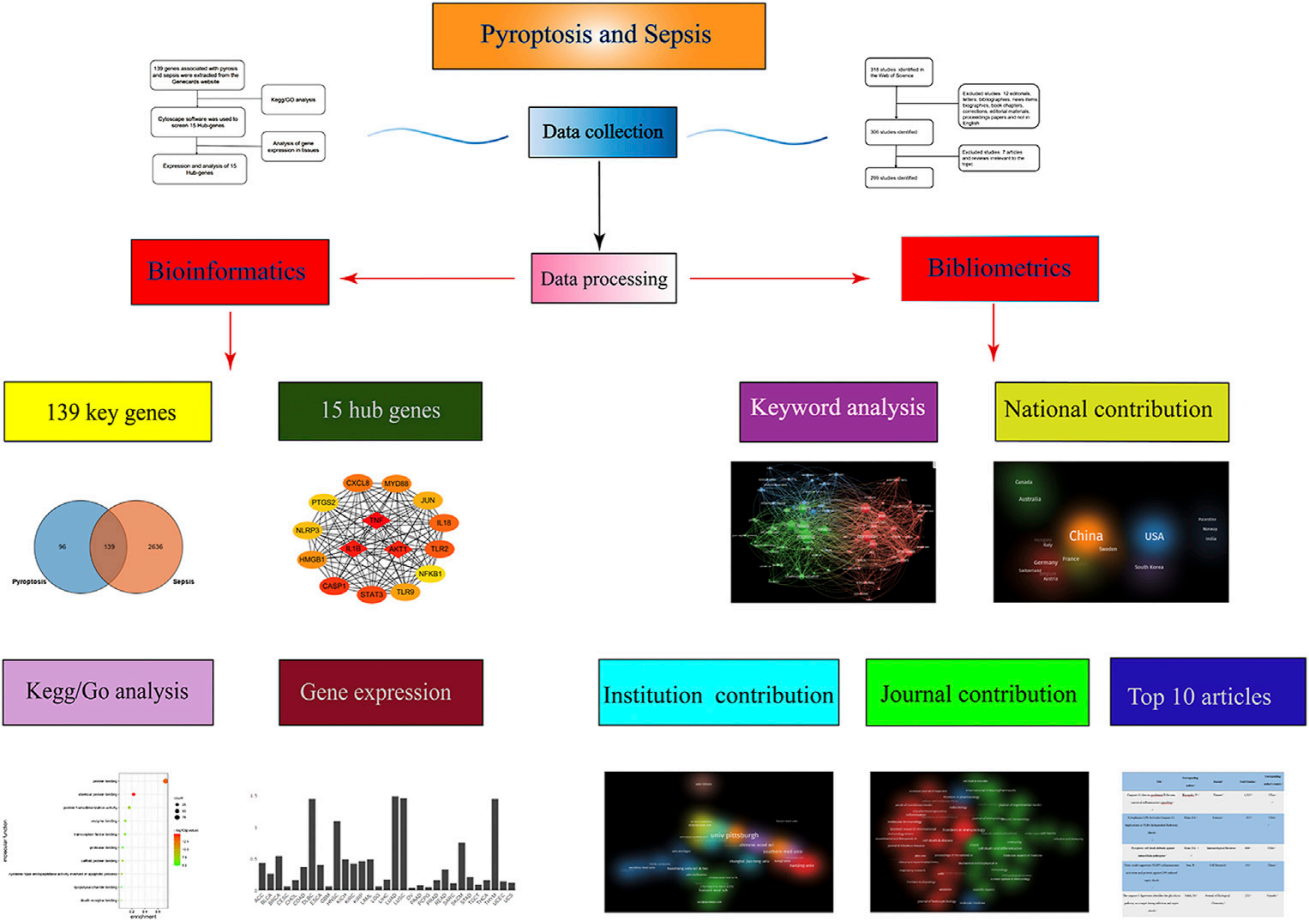
Bibliometrics and bioinformatics analysis flow chart of pyroptosis in sepsis studies.

For bibliometrics content, from 2012 to 2021, there were 318 articles related to the topic, 306 remained after limiting the type of articles and limiting the language to English, and 299 that met our inclusion criteria remained after screening (Figure 1). By analyzing and aggregating the data, the results will show the contribution of different journals, contribution of different countries, contribution of different institutions, and the top 10 articles in each related field. The specific process of the six aspects of contribution keywords of different institutions and contributions from related fields was shown in Figure 3. In order to avoid changes in the rank of genes caused by daily updates to the database (Figure 2A), the 139 genes most related to sepsis and pyroptosis were screened on 4 January 2022, and KEGG/GO analysis was conducted on the 139 genes (Figure 2B), analyzing the cellular components, molecular functions, biological processes, and signal pathways involved in iron death. We used CytoScope software to calculate and screen 15 hub genes from the 139 genes. Based on bibliometrics prediction of future development, 15 hub genes were applied to tissue expression. Specific results are shown in Figure 3.

### Bibliometrics content

**FIGURE 4:**
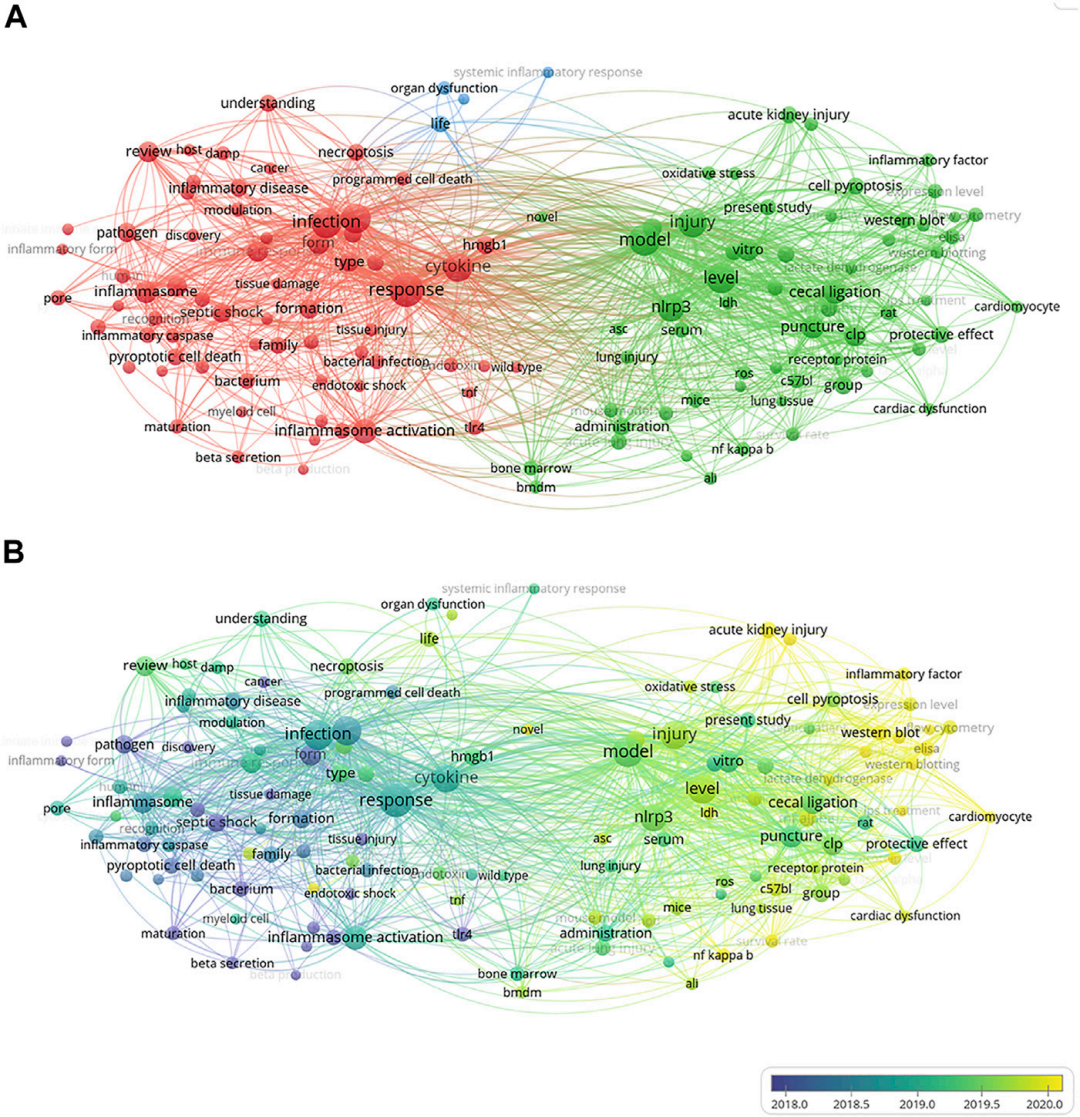
The co-occurrence analysis of all keywords in publications of pyroptosis in sepsis. **(A)** Mapping of the keywords in the area of pyroptosis in sepsis. The words were divided into three clusters in accordance with different colors generated by default: “animal research” (right in green), “cell research” (left in red), and “molecular research” (up in blue). The size of the circle represented the frequency of keywords; **(B)** The distribution of keywords was presented according to the average time of appearance. The blue color represented early appearance, and the yellow color stood for late appearance. Two keywords were considered co-occurred if they both occurred on the same line in the corpus file. A smaller distance between two keywords indicates relatively higher co-occurrences of the keyword.

Overall, 299 articles met our inclusion criteria, with China ranking first in the number of publications at 173 (54.4%) (Supplementary Figure S1). Considering the number of all-field publications, RRI values have risen (Supplementary Figure S1). Supplementary Figure S2 shows that China has the closest communication with the United States. As shown in Supplementary Figure S3, the University of Pittsburgh in the U.S. published the most papers among institutions worldwide, with 24 publications, representing 8.03% of all publications. The number of papers published in *Frontiers in Immunology* (IF = 7.561) was the highest with 16 records. In terms of impact factors, *Immunity* (IF = 31.745) was ranked first. The most cited article about pyroptosis in sepsis in the world is caspase-11 cleaves gasdermin D for non-canonical inflammasome signaling, which was written by Kayagaki, N in the USA, and this article has been cited 1,325 times (Supplementary Table S1). Among the most important parts of a paper are keywords. Analysis of keywords provides a summary of research topics in a field and explores hotspots and various directions for future research. Utilizing VOSviewer, we reviewed the keywords extracted from 299 publications. As presented in Figure 4A, in the process of the analysis, 130 keywords, defined as terms appearing at least nine times throughout the title and abstracts of all papers, were identified and grouped into three clusters: “animal research,” “cell research,” and “molecular research.” Figure 4A shows the results of a co-occurrence analysis of all incorporated keywords. As shown in Figure 4B, the blue color indicates that the word appeared relatively early in the research stage, while the yellow color indicates a more recent appearance. For example, “liver injury” (cluster one, animal research) has an AAY of 2,020.2 in the early stages of pyroptosis research and could be a new target. Among the first cluster of topics, “mir” and “aki” (cluster one, appearing 15 times and 13 times) with an AAY of 2,020.6 and 2,020.4, respectively, were noted as new topics. In the second cluster, “monocyte” (cluster two, cell research), with an AAY of 2,019.5, was the most recently emerging words, which appeared 15 times. Among the third cluster, “neutrophil” (cluster three, molecular research) was the most recent word, with an AAY of 2,019.7, which occurred 15 times.

### Functional enrichment analysis and expression of the top five hub genes

**FIGURE 5:**
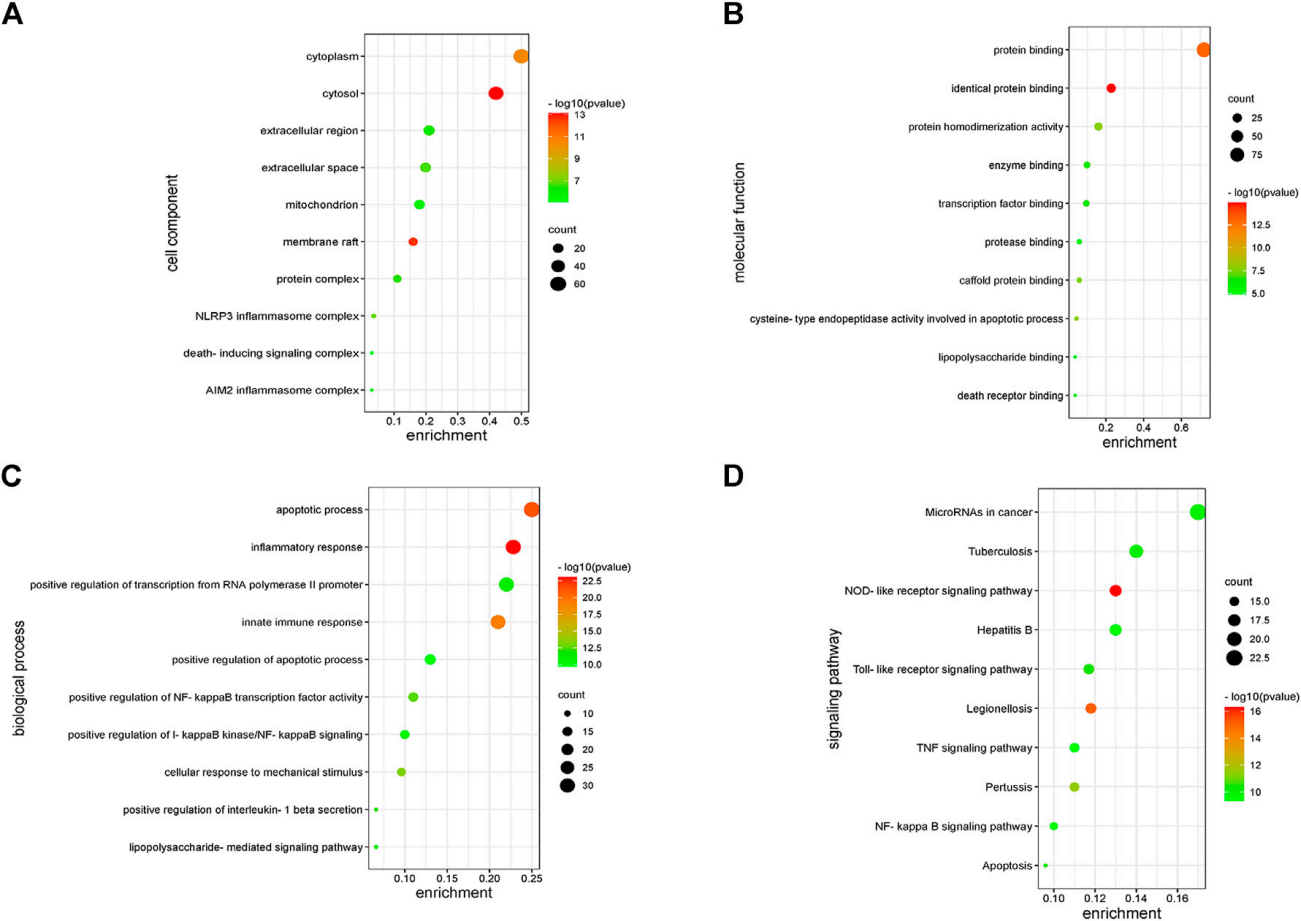
Enrichment analysis of key genes. **(A)** The enriched GO terms in the component (CC). **(B)** The enriched GO terms in the molecular function (MF). **(C)** The enriched GO terms in the biological process (BP). **(D)** KEGG enrichment analysis.

**FIGURE 6:**
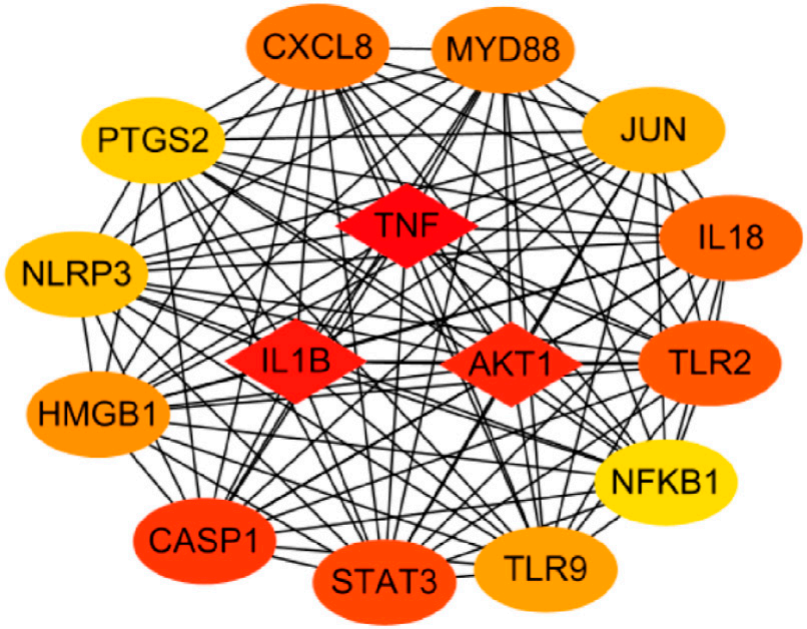
The top 15 hub genes. The network of the top 15 hub genes by CytoScope.

**FIGURE 7:**
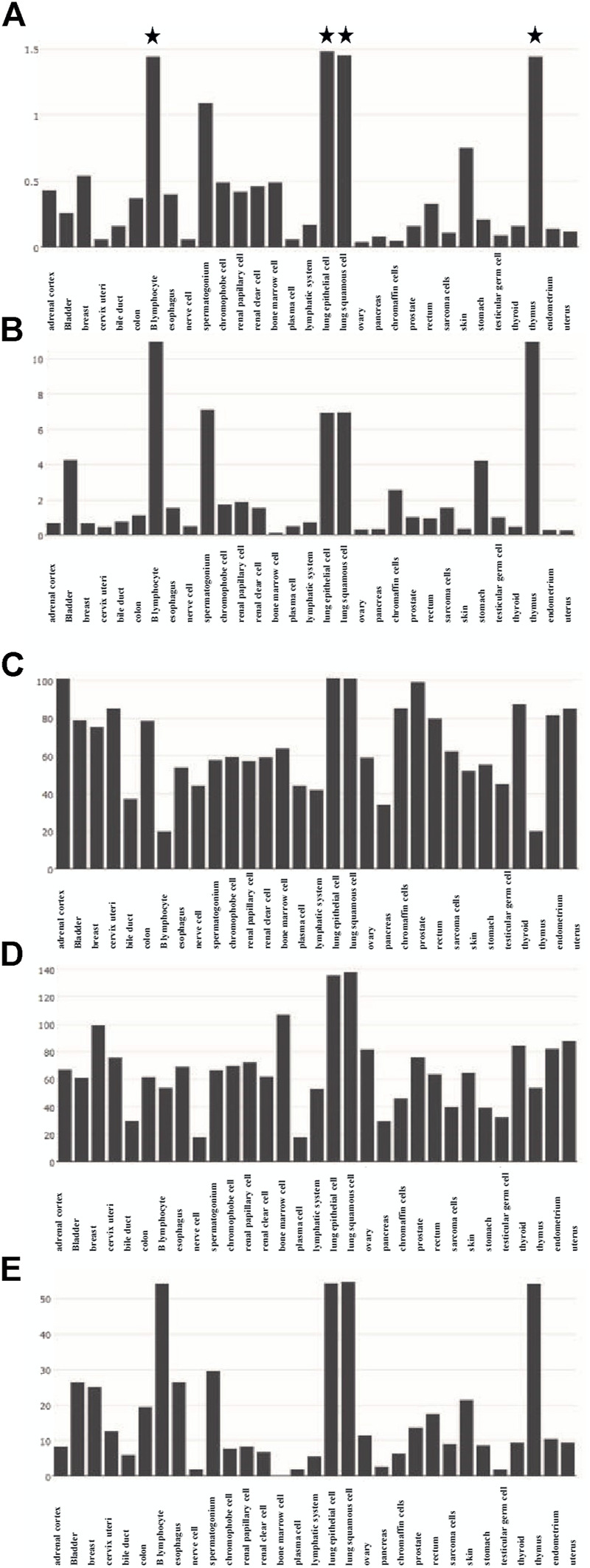
Expression of five hub genes in different tissues. **(A)** Expression of TNF in different tissues. **(B)** Expression of IL-1Β in different tissues. **(C)** Expression of AKT1 in different tissues. **(D)** Expression of CASP1 in different tissues. **(E)** Expression of STAT3 in different tissues.

The functions of 139 genes were further explored by GO enrichment analysis and KEGG pathway enrichment analysis. The results of GO enrichment analysis indicated that key genes that play a role in sepsis are mainly involved in biological processes such as inflammatory response, apoptotic, and innate immune response, and they play an identical protein-binding role in cell membranes and membrane rafts (Figures 5A–C). KEGG pathway analysis revealed that the predicted genes were mainly closely related to microRNAs in cancer, the hepatitis B pathway, and the NOD-like receptor signaling pathway (Figure 5D). Subsequently, a network of 15 hub genes was constructed and visualized by Cytoscape (Figure 6). For the screened hub genes, we selected the top five genes (TNF, IL-1β, AKT1, CASP1, and STAT3) sorted by Cytoscape software, and these five genes were exactly the key to the hub gene network. We used the gene expression website (http://gepia.cancer-pku.cn/) to evaluate the expression levels of these five genes in different tissues, and the results indicated that the expression levels of these five genes were highly expressed in lung tissues, thymus tissues, and lymphocytes (Figure 7).

## Discussion

### Research trends of pyroptosis in sepsis

There has been a steady increase in the number of studies on pyroptosis in sepsis based on the volume of publications. The study of pyroptosis in sepsis can be divided into three phases: “infancy” (2011–2014), “outbreak” (2014–2017), and “rapid development” (2017–2021). In view of the development trend, the study of pyroptosis in sepsis remains a subject for future studies and has huge development potential. In its infancy, pyroptosis has been poorly studied in sepsis, and at this time, research on pyroptosis is gradually being refined. In 2013, Dixit VM’s team published a paper in Science demonstrating that gram-negative bacteria can activate caspase-11 in cells by using LPS independent of the traditional LPS extracellular receptor TLR4 (Kayagaki et al., 2013). However, the mechanism of LPS-activated caspase-11 has not been fully elucidated. However, with the further research in this field, it has been proved that berberine alkaloids (Yuan et al., 2021), bacterial endotoxin (Yang et al., 2019), and heparin (Tang et al., 2021) all affect the coke decay of cells by affecting caspase-11. During the outbreak period, research on pyroptosis in sepsis proliferated, which showed 4/5/11 can bind LPS produced by intracellular bacteria without the need for other intracellular receptors, which can directly activate caspase-11 without the action of caspase-1 and then cause pyroptosis (Shi et al., 2014). In 2015, Shao Feng’s group and the Dixit VM’s team published papers in *nature*, respectively, to clarify that GSDMD is the common substrate for activated caspase-4 and caspase-11 (Kayagaki et al., 2015; Shi et al., 2015). In 2017, the work of Shao Feng et al. further confirmed that the gasdermin family is the ultimate effector protein that directly triggers the pyroptosis process (Ding et al., 2016). The research on pyroptosis in sepsis has also ushered in a period of rapid development, and a large number of literature studies have emerged (Supplementary Figure S1).

From a distribution point of view, pyroptosis research in sepsis is mostly conducted in China and the United States (Figure 4), which may be because the research on pyroptosis in China and the United States started earlier, with sufficiently influential research institutions and leading figures. In addition, the cooperation and exchanges between China and the United States are close, which also promotes the development of research to a certain extent (Scarazzati and Wang, 2019). In an era of globalization, the promotion of research on pyroptosis in sepsis requires cooperation and coordination between countries. Accounting for 2.1%, immunity (if = 31.745) is the influential journal in the field (Supplementary Table S1). Half of the top ten cited papers are from the United States, which is consistent with the results in Supplementary Figure S1.

### Research focused on pyroptosis in sepsis

Cellular pyroptosis relies on cysteine-aspartate proteases (caspase) to activate programmed cell death patterns. It is characterized by micropores in the cell membrane, swelling and rupture of cells, release of cell contents, secretion of inflammatory factors, and promotion of innate immunity and cell death. Inflammasome activation and cytokine storm play an important role in the development of sepsis. Pyroptosis also destroys infected cells and promotes the release of pathogens to be phagocytosed and killed by immune cells, thus promoting the presentation of antigens and the elimination of intracellular pathogens. Moderate pyroptosis can protect the body against pathogen infection, but excessive activation of pyroptosis may aggravate sepsis and septic shock.

Through co-occurrence analysis of key words, three groups were obtained, namely, animal research, cell research, and molecular research (Figure 4A). Among them, we can find that the current research direction is shifting from the exploration of the mechanism of pyroptosis to the application of pyroptosis in sepsis (Figure 4B). Among research hotspots, liver injury (cluster one) in animal research is the latest. Experiments showed that hepatic cell pyroptosis was correlated with the degree of septic liver injury, and pyroptosis-related proteins such as caspase-1 and NLRP3 significantly increased. In addition, the application of pyroptosis-related protein inhibitors can also alleviate liver damage (Chen et al., 2016). The excessive activation of hepatocyte pyroptosis can cause the release of a large number of inflammatory cytokines, such as IL-6, TNF-α, and IL-1β, further aggravating liver cell damage. In 2001, Menzel et al. (2011) found that caspase-1 can alleviate liver injury and inflammatory response; Zhao et al. (2016) found that activated NLRP1 and NLRP3 can promote the secretion of IL-1β. These results indicate that caspase-1, NLRP3, and NLRP1 play an important role in sepsis. Cao et al. (2015) used caspase-1 inhibitor, AC-YVAD-CMK, to reduce CLP-induced acute kidney injury, which may provide target in the treatment of the sepsis. As the activation form of cell pyroptosis, caspase-1 and caspase-11 are the core points. Pyroptosis is GSDMD-pore–induced cell swelling and rupture, and caspase-1 activated maturation and secretion of inflammatory factors such as IL-1β and IL-18: these are the characteristic manifestations of pyroptosis. The activation mechanism of caspase-11 is completely different from that of caspase-1. The activation of caspase-11 does not require the participation of the ASC gene. At present, the specific mechanisms of caspase-11 destroying cell integrity, inducing cell pyroptosis, and regulating inflammatory response need further study. In the second and third clusters, monocyte is the most recently emerging word. In sepsis, innate immunity is the first line of defense against invading pathogens. In the early stage of sepsis, monocytes can not only prevent the increase of pathogenic bacteria by recognizing and intercepting antigens, but can also differentiate into macrophages to play an immunomodulatory role. However, induction of pyroptosis in monocytes causes a systemic inflammatory cascade that ultimately leads to tissue and organ damage (Oliva-Martin et al., 2016). These keywords appear in hotspots and represent the latest research directions.

### Analysis of gene functional enrichment and hub genes

KEGG pathway analysis identified significant enrichment of NOD-like receptor signaling pathways related to the immune system (*p* < 0.05). Oligomerization nucleotide-binding domain–like receptors (NLRs) are a family of cytoplasmic proteins that are crucial in intracellular ligand recognition. These NLRs are responsible for recognizing specific pathogen molecules or host-derived damage signals in the cytoplasm and triggering innate immune responses. NLRP3 and NLRP1 are also members of the NLR family. Among these, the NLRP3 inflammasome is the most extensively studied inflammasome. The NLRP3 inflammasome promotes the maturation and secretion of pro-inflammatory cytokines and triggers pyroptosis after activation (Schroder and Tschopp, 2010). Consequently, inhibiting the NLRP inflammasome has emerged as an attractive therapeutic target for sepsis. Protein-protein–related interaction analysis was performed on key genes, and most of the encoded proteins have interaction relationships. By further analysis of the network, 15 hub genes were obtained as follows. These genes are mainly involved in the cytokine–cytokine receptor interaction pathway (TNF, IL-1Β, IL-18, and CXCL8), toll-like receptor signaling pathway (TLR2, TLR9, and MYD88), NOD-like receptor signaling pathway (NLRP3), etc. Cytokines associated with sepsis, including interleukins, tumor necrosis factor, chemokines, high-mobility group proteins, and other molecules, play a key role in the inflammatory process of sepsis. ILs are the most important cytokines released during infection; they initiate signaling and promote the activation, proliferation, migration, and necrosis of immune cells. Interleukin 1β (IL-1β) is a key mediator of the body’s response to inflammation with multiple biological effects, and its deficiency can lead to a variety of diseases. IL-1β is mainly produced by monocytes and macrophages in innate immune system cells, and its biosynthesis and release are regulated by pathogen-associated molecular patterns (PAMPs) and damage-associated molecular patterns (DAMPs). PAMP/DAMP induces activation of the NLRP3 inflammasome in macrophages and production of pro-inflammatory cytokine—IL-1B, thus promoting sepsis (Hughes et al., 2014). There is substantial evidence that a series of treatments of monocytes, macrophages, and dendritic cells with LPS and ATP induce rapid and efficient releases of IL-1β (Molteni et al., 2018). Interleukin-18 (IL-18) is a pro-inflammatory cytokine that is encoded in humans by the IL-18 gene. Many cell types, including hematopoietic and non-hematopoietic cells, have the potential to produce IL-18 (Wawrocki et al., 2016). Excessive production of IL-18 has been associated with critical illnesses, including myocardial ischemia and acute kidney injury. The higher the serum IL-18 level, the more severe the liver and kidney function and pathological injury, which is considered as a potential biomarker for sepsis patients. Previous studies have shown that NLRP3 inflammatory vesicles also regulate the release of IL-18 (Nakahira et al., 2011). The pathogen-associated molecular patterns and damage-associated molecular patterns in sepsis activate NLRP3 and caspase-1 to release inflammatory cytokines such as IL-18 and IL-1β, aggravating the inflammatory response (Abderrazak et al., 2015). Toll-like receptors (TLRs) are members of the pattern recognition receptors (PRRs) that contribute to identifying pathogenic microorganisms, mediating cellular signaling systems, and releasing inflammatory factors. TLR4 is the principal receptor that recognizes the lipopolysaccharide of the outer cell wall of gram-negative bacteria, and knockdown of the TLR4 gene helps to defend against endotoxemia (Luo et al., 2012). This is due to the fact that activated TLR4 causes an excessive inflammatory response, which in turn leads to a systemic inflammatory response. In summary, nucleotide-binding oligomerization domain–like receptors (NLRs) are a family of 23 receptors that play a key role in the regulation of the host innate immune response. Several studies have found that NLRP3 inflammatory vesicles play a key role in the development and progression of sepsis, and more studies have found inhibiting NLRP3 to be an effective treatment for sepsis (Guo et al., 2015; Piippo et al., 2018). TLRs, along with the NOD signaling pathway, enable the integration of multiple signal pathways as a network that plays a synergistic role in the infection and immunity of sepsis.

## Conclusion

We expect the total number of global publications to grow, according to RRI. Importantly, our analysis shows that animal studies will be the subject of this field in the future, and liver injury, AKI, monocytes, and neutrophils may be hotspots for future research. The 139 genes associated with pyroptosis and sepsis play a crucial role in the expression of microRNAs in the cancer pathway, hepatitis B pathway, and NOD-like receptor signaling pathway. Hub genes such as TNF, IL-1Β, AKT1, CASP1, and STAT3 play a central role in this study, and they are highly expressed in lung tissues, thymus tissues, and lymphocytes.

## Data Availability

The original contributions presented in the study are included in the article/Supplementary Material; further inquiries can be directed to the corresponding authors.
