# Supplementary material for: Pyroptosis in sepsis: Comprehensive analysis of research hotspots and core genes in 2022
Source: Front Mol Biosci. 2022 Aug 11;9:955991. doi: 10.3389/fmolb.2022.955991 (PMC9402944; doi:10.3389/fmolb.2022.955991)
Supplement: Supplementary file 1 [file Table1.DOCX]

| **Title** | **Corresponding authors** | **Journal** | **Total Citations** | **Corresponding**  **author’s country** |
| --- | --- | --- | --- | --- |
| Caspase-11 cleaves gasdermin D for non-canonical inflammasome signalling | Kayagaki, N | Nature | 1,325 | USA |
| Cytoplasmic LPS Activates Caspase-11: Implications in TLR4-Independent Endotoxic Shock | Miao, EA | Science | 657 | USA |
| Pyroptotic cell death defends against intracellular pathogens | Miao, EA | Immunological Reviews | 400 | USA |
| Nitric oxide suppresses NLRP3 inflammasome activation and protects against LPS-induced septic shock | Sun, B | Cell Research | 240 | China |
| The caspase-1 digestome identifies the glycolysis pathway as a target during infection and septic shock | Saleh, M | Journal of Biological Chemistry | 232 | Canada |
| Caspase-11 Requires the Pannexin-1 Channel and the Purinergic P2X7 Pore to Mediate Pyroptosis and Endotoxic Shock | Liu, Q | Immunity | 222 | China |
| High Mobility Group Box Protein 1 (HMGB1): The Prototypical Endogenous Danger Molecule | Yang, H | Molecular Medicine | 181 | USA |
| The Endotoxin Delivery Protein HMGB1 Mediates Caspase-11-Dependent Lethality in Sepsis | Lu, B | Immunity | 171 | China |
| Extracellular histones in tissue injury and inflammation | Anders, HJ | Journal of Molecular Medicine-Jmm | 170 | Germany |
| Inflammasomes | Flavell, RA | Cold Spring Harbor Perspectives in Biology | 166 | USA |
