# Supplementary figures and images for: Pyroptosis in sepsis: Comprehensive analysis of research hotspots and core genes in 2022
Source: Front Mol Biosci. 2022 Aug 11;9:955991. doi: 10.3389/fmolb.2022.955991 (PMC9402944; doi:10.3389/fmolb.2022.955991)

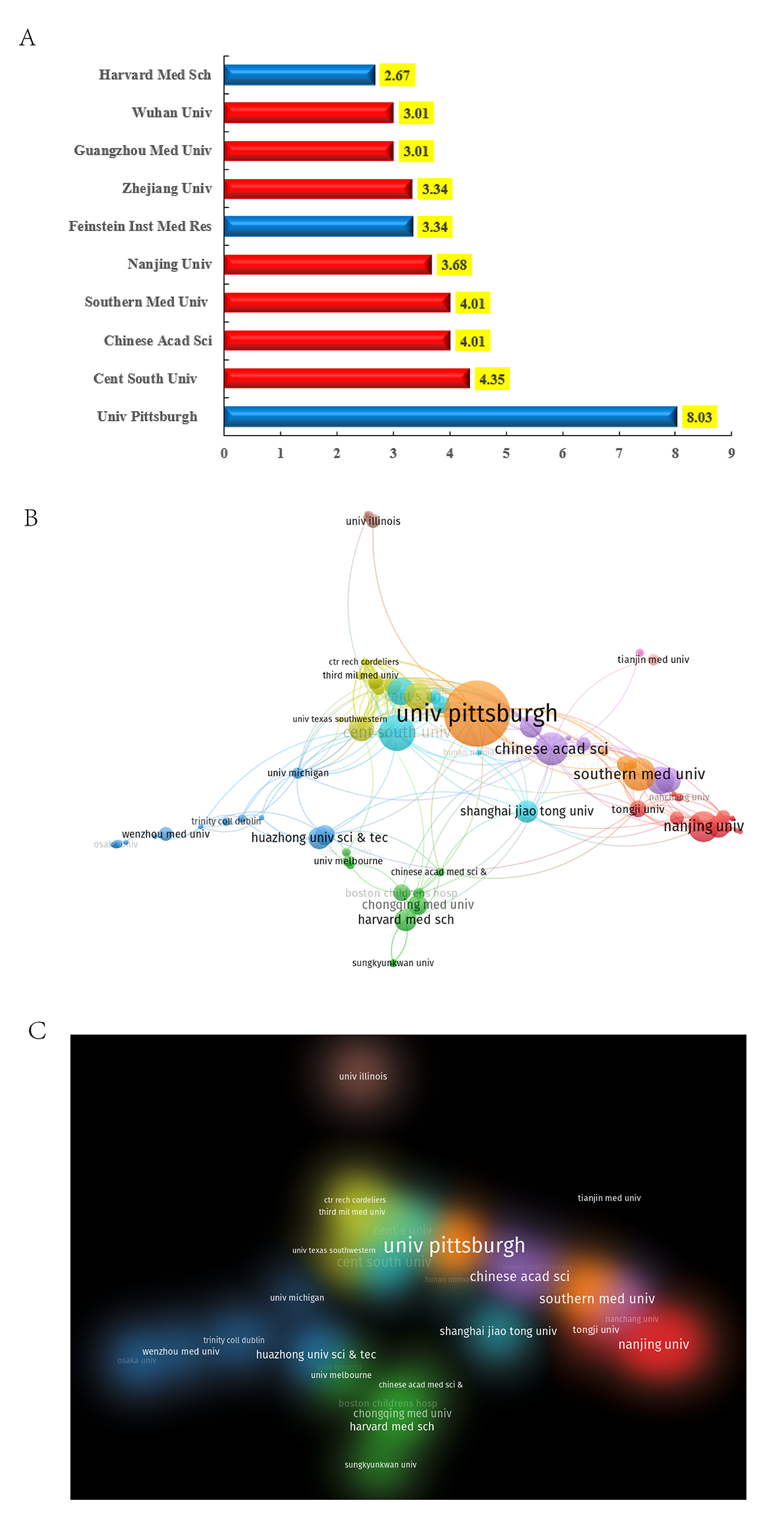

Supplement: Supplementary file 2 [file Image3.TIF]

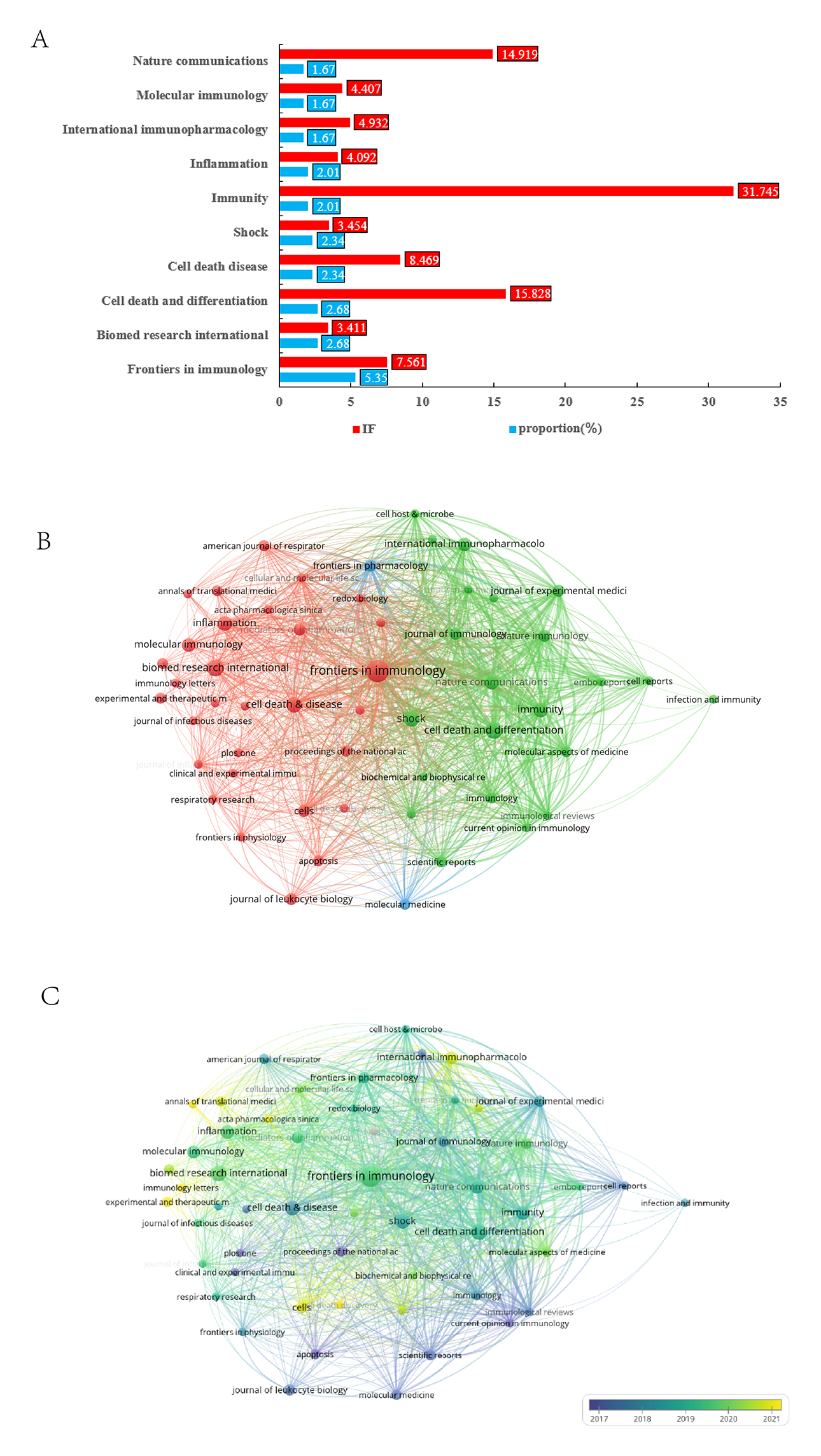

Supplement: Supplementary file 3 [file Image4.TIF]

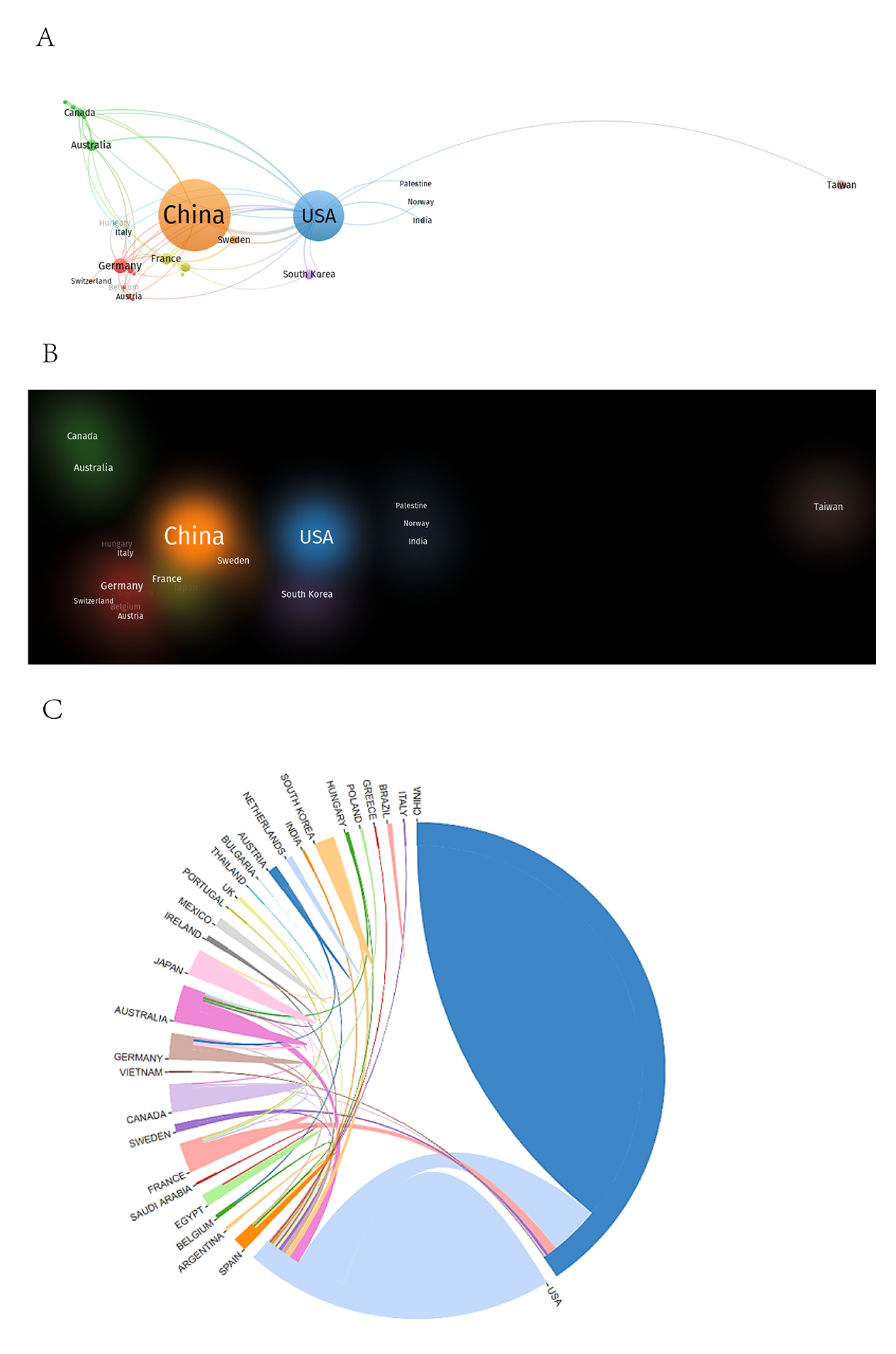

Supplement: Supplementary file 4 [file Image2.TIF]

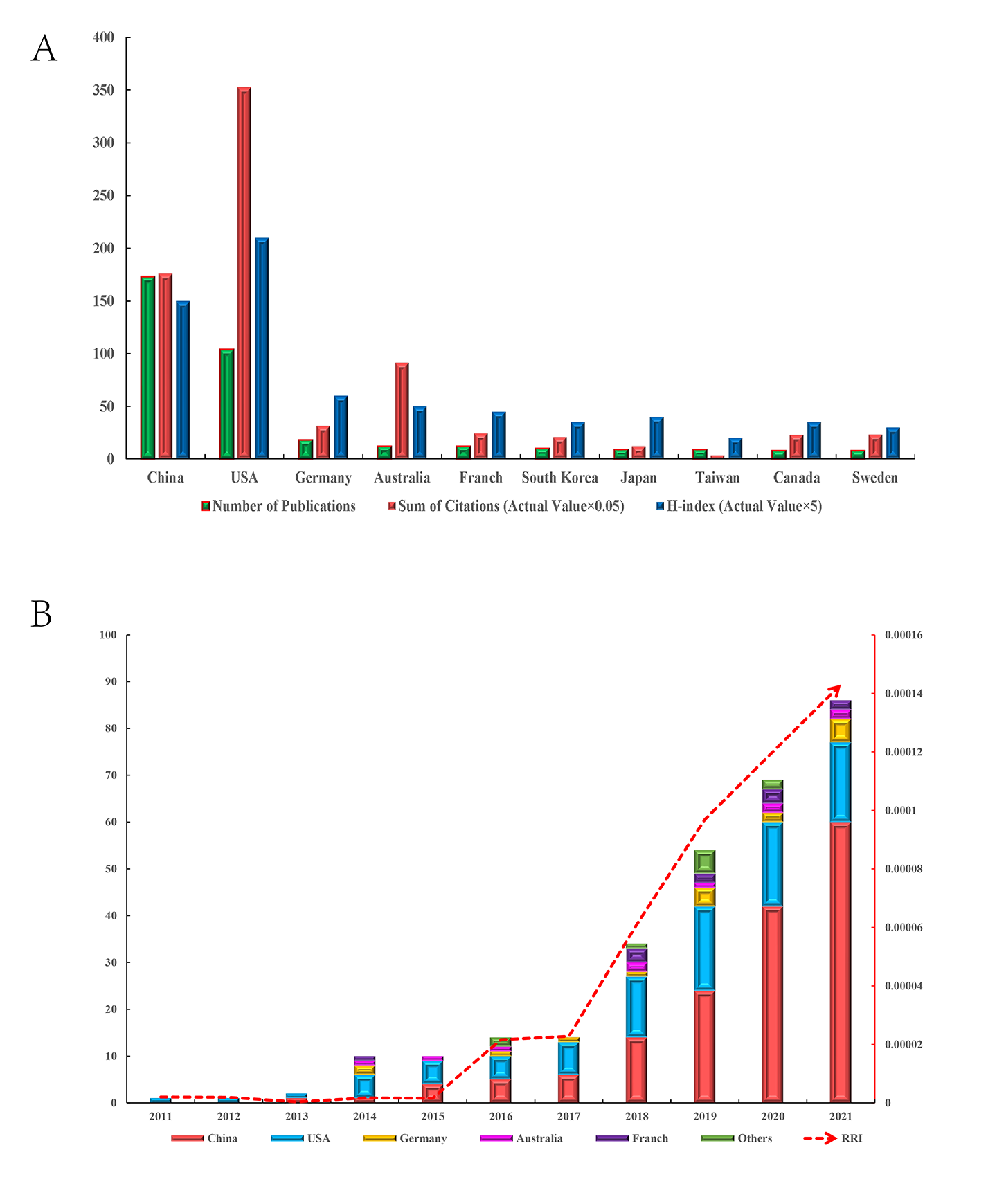

Supplement: Supplementary file 5 [file Image1.TIF]
