# Supplementary material for: Pyroptosis in sepsis: Comprehensive analysis of research hotspots and core genes in 2022
Source: Front Mol Biosci. 2022 Aug 11;9:955991. doi: 10.3389/fmolb.2022.955991 (PMC9402944; doi:10.3389/fmolb.2022.955991)
Supplement: Supplementary file 6 [file Table2.DOCX]

**Supplemental Table 1. The analytic consequence of 90 keywords with at least 9 occurrence times**

| **Label** | **Cluster** | **Occurrences** | **Average appearing years  (AAY)** | |
| --- | --- | --- | --- | --- |
| bone marrow | 1 | 19 | | 2019.1053 |
| rat | 1 | 18 | | 2019.1111 |
| protective effect | 1 | 24 | | 2019.1667 |
| present study | 1 | 21 | | 2019.1905 |
| lung injury | 1 | 15 | | 2019.2 |
| puncture | 1 | 47 | | 2019.2553 |
| vitro | 1 | 43 | | 2019.2558 |
| serum | 1 | 21 | | 2019.2857 |
| acute lung injury | 1 | 21 | | 2019.3333 |
| model | 1 | 97 | | 2019.3918 |
| alpha | 1 | 30 | | 2019.4 |
| cecal ligation | 1 | 47 | | 2019.4255 |
| expression | 1 | 112 | | 2019.4375 |
| nlrp3 | 1 | 71 | | 2019.4789 |
| clp | 1 | 43 | | 2019.4884 |
| progression | 1 | 18 | | 2019.5 |
| ros | 1 | 17 | | 2019.5294 |
| cell pyroptosis | 1 | 23 | | 2019.5652 |
| group | 1 | 29 | | 2019.5862 |
| bmdm | 1 | 13 | | 2019.6154 |
| lung | 1 | 19 | | 2019.6316 |
| mice | 1 | 20 | | 2019.65 |
| level | 1 | 83 | | 2019.6627 |
| ali | 1 | 12 | | 2019.6667 |
| nod | 1 | 17 | | 2019.7059 |
| control | 1 | 31 | | 2019.7097 |
| liver | 1 | 14 | | 2019.7143 |
| asc | 1 | 11 | | 2019.7273 |
| septic mouse | 1 | 19 | | 2019.7368 |
| mouse model | 1 | 16 | | 2019.75 |
| oxidative stress | 1 | 14 | | 2019.7857 |
| c57bl | 1 | 19 | | 2019.7895 |
| ldh | 1 | 16 | | 2019.8125 |
| tnf alpha | 1 | 24 | | 2019.8333 |
| expression level | 1 | 16 | | 2019.875 |
| flow cytometry | 1 | 16 | | 2019.875 |
| protein level | 1 | 15 | | 2019.9333 |
| elisa | 1 | 19 | | 2020.0526 |
| acute kidney injury | 1 | 22 | | 2020.1818 |
| liver injury | 1 | 18 | | 2020.2222 |
| nf kappa b | 1 | 15 | | 2020.3333 |
| aki | 1 | 15 | | 2020.4 |
| beta secretion | 2 | 13 | | 2016.7692 |
| toll | 2 | 20 | | 2017.45 |
| septic shock | 2 | 31 | | 2017.6452 |
| maturation | 2 | 16 | | 2017.875 |
| innate immunity | 2 | 19 | | 2017.8947 |
| tlr4 | 2 | 19 | | 2017.8947 |
| bacterium | 2 | 21 | | 2018 |
| pathogen | 2 | 30 | | 2018.0667 |
| pyroptotic cell death | 2 | 22 | | 2018.1818 |
| secretion | 2 | 37 | | 2018.1892 |
| inflammatory caspase | 2 | 20 | | 2018.2 |
| form | 2 | 46 | | 2018.2174 |
| induction | 2 | 17 | | 2018.2941 |
| cytosolic lps | 2 | 12 | | 2018.3333 |
| formation | 2 | 31 | | 2018.3548 |
| programmed cell death | 2 | 17 | | 2018.4706 |
| bacterial infection | 2 | 19 | | 2018.4737 |
| gram negative bacterium | 2 | 18 | | 2018.5 |
| inflammasome | 2 | 42 | | 2018.5476 |
| cell death | 2 | 85 | | 2018.5647 |
| cytosol | 2 | 17 | | 2018.5882 |
| infection | 2 | 92 | | 2018.75 |
| response | 2 | 141 | | 2018.8652 |
| cytokine | 2 | 78 | | 2018.8718 |
| human | 2 | 18 | | 2018.8889 |
| inflammasome activation | 2 | 52 | | 2018.9038 |
| cleavage | 2 | 32 | | 2019.0625 |
| release | 2 | 95 | | 2019.1684 |
| type | 2 | 38 | | 2019.2632 |
| endotoxin | 2 | 17 | | 2019.4118 |
| monocyte | 2 | 15 | | 2019.5333 |
| inflammatory disease | 3 | 25 | | 2018.52 |
| inflammatory cell death | 3 | 15 | | 2018.7333 |
| molecular pattern | 3 | 17 | | 2018.9412 |
| development | 3 | 46 | | 2019.087 |
| condition | 3 | 30 | | 2019.1333 |
| damp | 3 | 15 | | 2019.1333 |
| organ dysfunction | 3 | 15 | | 2019.1333 |
| process | 3 | 39 | | 2019.1538 |
| understanding | 3 | 23 | | 2019.1739 |
| molecule | 3 | 30 | | 2019.2 |
| high mobility group box | 3 | 22 | | 2019.2727 |
| hmgb1 | 3 | 28 | | 2019.2857 |
| review | 3 | 36 | | 2019.3611 |
| necroptosis | 3 | 23 | | 2019.4783 |
| pathogenesis | 3 | 30 | | 2019.6333 |
| life | 3 | 22 | | 2019.6364 |
| neutrophil | 3 | 15 | | 2019.7333 |
